# Melatonin Rhythm and Its Relation to Sleep and Circadian Parameters in Children and Adolescents With Autism Spectrum Disorder

**DOI:** 10.3389/fneur.2022.813692

**Published:** 2022-06-14

**Authors:** Elena Martinez-Cayuelas, Teresa Gavela-Pérez, María Rodrigo-Moreno, Milagros Merino-Andreu, Claudia Vales-Villamarín, Iris Pérez-Nadador, Carmen Garcés, Leandro Soriano-Guillén

**Affiliations:** ^1^Department of Pediatrics, Instituto de Investigaciones Sanitarias- Fundación Jiménez Díaz, Universidad Autónoma de Madrid, Madrid, Spain; ^2^Hospital Universitario La Paz, Paseo de la Castellana, Madrid, Spain; ^3^Lipid Laboratory, Instituto de Investigaciones Sanitarias-Fundación Jiménez Díaz, Universidad Autónoma de Madrid, Madrid, Spain

**Keywords:** melatonin, autism spectrum disorder, slee, circadian rhythm, actigraphy, puberty

## Abstract

**Introduction:**

Sleep problems are prevalent among individuals with autism spectrum disorder (ASD), and a role has been attributed to melatonin in this multifactorial comorbidity.

**Methods:**

A cross-sectional study was conducted on 41 autistic children and adolescents (9.9 ± 3.02) and 24 children and adolescents with a normal intellectual function (8.42 ± 2.43) were used as controls. Subjects were matched for sex, body mass index, and pubertal stage, and all were drug-naive. Circadian and sleep parameters were studied using an ambulatory circadian monitoring (ACM) device, and saliva samples were collected around the onset of sleep to determine dim light melatonin onset (DLMO).

**Results:**

Prepubertal individuals with ASD presented later DLMO and an earlier decline in melatonin during adolescence. A relationship was found between melatonin and both sleep and circadian parameters. Participants and controls with later DLMOs were more likely to have delayed sleep onset times. In the ASD group, subjects with the later daytime midpoint of temperature had a later DLMO. Later melatonin peak time and DLMO time were related to lower general motor activity and lower stability of its rhythms.

**Conclusion:**

The melatonin secretion pattern was different in individuals with ASD, and it showed a relationship with sleep and circadian parameters. Alterations in DLMO have not been previously reported in ASD with the exception of more variable DLMO timing; however, high variability in the study design and sample characteristics prevents direct comparison. The ACM device enabled the measurement of circadian rhythm, a scarcely described parameter in autistic children. When studied in combination with other measures such as melatonin, ACM can offer further knowledge on sleep problems in ASD.

## Introduction

Sleep problems (SPs) are prevalent in children with autism spectrum disorder (ASD) and mostly consist of alterations in sleep onset and sleep maintenance difficulties. SPs can impair social and cognitive function, which reduces the quality of life (1–3). Furthermore, sleep and behavior can affect each other in a bidirectional way, and it is known that comorbid mental health conditions, such as anxiety, depression, and attention-deficit hyperactivity disorder, can impair sleep themselves (4–6). Although the causes of SPs in ASD are diverse, it has been postulated that misalignment of the circadian rhythm, in which melatonin acts as the main regulator, is involved in the etiology (1, 7).

Melatonin is a neurohormone that is mainly produced nocturnally in the pineal gland by the conversion of serotonin to N-acetylserotonin (NAS). Melatonin is crucial to circadian rhythm regulation, including sleep–wake cycles and neuroendocrine and body temperature rhythms; nighttime melatonin concentration is typically at least three-fold of daytime values and is expected to peak around 2 a.m. (8, 9). The production of melatonin conveys the message of darkness and induces night-state physiological functions; it increases peripheral blood flow in humans, thus lowering the core temperature, which is associated with sleep onset (10–12).

Measures of melatonin are considered the most relevant peripheral index of human circadian rhythmicity. Saliva samples have been proposed as the most practical and reliable method of assessing the circadian phase as research has found a high correlation coefficient between plasma and salivary levels (13). Dim light melatonin onset (DLMO) is defined as the time at which a salivary concentration of melatonin of 3-5 pg/ml is reached, which is expected to occur 2–3 h before sleep onset. This variable is considered a reliable marker of the circadian phase (9, 13, 14).

The study of sleep and circadian rhythmicity in neurodevelopmental disorders has increased over the years. Several studies (8, 15–17) and reviews (18, 19) have shown lower melatonin concentrations in autistic people, suggesting an overall deficit in melatonin production, at night and during daytime, which indicates that these individuals have reduced pineal and extrapineal production. (See Supplementary Table 1 for further information.) Moreover, nocturnal excretion of 6-sulfatoxymelatonin has been negatively correlated with the severity of ASD symptoms (8, 17), and dysregulation of biological pathways that maintain adequate levels of melatonin has been hypothesized to play an important role in sleep disturbances in ASD (20). By contrast, studies conducted by Goldman et al. in 2014 and 2017 (21, 22) reported normal overnight blood and salivary melatonin profiles in autistic children. Also, Braam et al. (23) reported elevated salivary melatonin levels (>50 pg/ml) and prolonged melatonin half-life (>5 h) in 15 intellectually disabled children and adolescents with sleep onset insomnia (including seven with ASD). Previous studies used different timelines and techniques to study the melatonin profile, which further complicates efforts to understand melatonin in ASD. In particular, research on DLMO in ASD is scarce (21, 22, 24) and does not allow one to draw final conclusions. Studies including other types of neurodevelopmental disorder, such as attention-deficit hyperactivity disorder (ADHD), have better defined the presence of a delayed DLMO, longer sleep latencies, and disruption of sleep maintenance (25–27).

To our knowledge, few research studies of autistic children and adolescents have explored evening salivary melatonin in a way that focuses on the relationship between this variable and sleep and circadian measures (22). The aim of this study was to define sleep and circadian patterns in children and adolescents with ASD using a combination of actigraphy recordings and measurements of saliva, comparing these measures against a sex-, body mass index-, and pubertal stage-matched control group. We hypothesized that autistic children and adolescents would have melatonin profiles different from typically developing (TD) controls, as well as prolonged sleep latencies. In contrast to prior research, we included only those participants who were not currently under any kind of pharmacological treatment so as to minimize the possible effect of drug therapy on sleep and circadian rhythms.

## Methods

### Study Design and Procedure

This cross-sectional study was conducted in the Department of Pediatrics of the University Hospital Fundación Jiménez Díaz, between September 2018 and January 2021.

### Sample Size Calculation

Assuming that the control group showed 5% of altered DLMO (advanced, delayed, or irregular DLMO) and this percentage was 20% in the ASD group, as reported in previous studies of children with developmental disorders (28), we determined that in order to reach a β-statistical power of 80% and a level of α significance of 5%, at least 24 individuals would be needed in each group. Data and Supplemental Materials are available through the Open Science Framework (https://osf.io/jkb24/).

### Participants

The sample described in this article comprises a subset of individuals included in a previous study, and details of participant recruitment and study design are described in a prior publication (29). Briefly, children and adolescents between 5 and 18 years of age who met the clinical criteria for ASD as confirmed by the Autism Diagnostic Observation Schedule (30) were recruited from pediatric neurology clinics. Only those with adequate saliva collection were included in this study. Those receiving any kind of medical treatment and those with attention-deficit hyperactivity disorder and specific genetic syndromes, such as fragile X, were excluded from the study as these disorders can associate impaired sleep themselves (25, 31, 32). Children and adolescents with intellectual disabilities were included. The control group was composed of TD children and adolescents between 5 and 18 years of age, with no mental or general disorder and under no pharmacological treatment, matched for sex, pubertal stage, and body mass index. Hospital workers were approached to find possible volunteers in their overall social environment (e.g., family, friends, and school classmates).

### Anthropometric Variables and Pubertal Stage

We collected data on BMI and pubertal stage using the criteria of Tanner and Whitehouse (33, 34).

### Ethics

The study protocol was approved by the local institutional review board. Parents of the participants provided written informed consent after the nature of the procedures had been fully explained. The study was performed in accordance with the principles of the Helsinki Declaration and the prevailing Spanish legislation on clinical research in human subjects.

### Ambulatory Circadian Monitoring (ACM)

Each participant wore an ACM device (Kronowise^®^) on their non-dominant wrist for 1 week (including one complete weekend), and all were asked to follow their usual routine. Specific data on the device and parameters can be found in a previous article (29). Of all raw data obtained from the ACM device, the specific variables described in this study were wrist temperature (WT, řC), motor activity (sum of accelerations from the three axes, expressed as G/h), time in movement (seconds), light exposure (including total light and blue light, measured as lux and log_10_lux), and sleep (converted into a binary code, with 1 corresponding to a resting period and 0 corresponding to an active period, for non-parametric index calculation). An integrated variable—known as thermometry, actimetry, and body position, TAP—is then obtained by integrating wrist temperature (inverted), motor activity, and position variability. TAP expresses general activation through arbitrary units (AU), where values near 1 indicate a high level of activation and values around 0 indicate complete rest (35). In addition, parents completed a 7-day, sleep–wake diary that was used as a backup for the ACM recordings if needed.

Circadian rhythms were analyzed through non-parametric analysis as described in a previous study for rest–activity data (36). This analysis was applied for each specific study variable yielding the following circadian parameters:

- Normalized relative amplitude (NRA): It quantifies the difference between the values shown by the variable during nighttime/sleep and the values shown during daytime/wake: the higher the NRA (range between 0 and 1), the better the consolidation of daytime activity and nighttime sleep. In the case of variables with an acrophase that occurred during the rest period (WT and sleep), the NRA was calculated based on the ratio of M5 (average measured for the 5 consecutive h with the maximum values) and L10 (average measured for the 10 consecutive h with the minimum values) across the averaged 24-h profile. Conversely, in the case of variables with an acrophase that occurs during the activity period (light exposure, activity, and position), these calculations were modified by using the 5 consecutive h of minimum values (L5) and the 10 consecutive h of maximum values (M10).
- Inter-daily stability (IS): it quantifies invariability day by day. IS values range from 0, for no stability, to 1, indicating that the pattern is repeated perfectly every single day.
- Intradaily variability (IV): It quantifies the degree of fragmentation of the behavioral rhythm within a day; its value ranges from 0, indicating that transitions of the specific variable within a day are tightly consolidated, to 2, which means that the fragmentation of transitions is random.
- Circadian function index (CFI): It is calculated as (IS+(2-IV) + NRA)/3, which assesses the global circadian rhythmicity status; CFI values range from 0 to 1, 1 being perfectly regular rhythms, unfragmented, and with high amplitude (37).

### Melatonin Sampling

Melatonin salivary collection was performed one night of the week in which children and adolescents wore the ACM device. Hourly samples were taken: 3, 2, and 1 h before bedtime; to record any continued rise in melatonin concentrations, additional measurements were taken at bedtime and 1 h after bedtime. This timing allowed for the determination of DLMO described to occur 2 to 3 h before habitual sleep onset time (13, 14). The participants used saliva collection tubes. As mentioned in the literature, during the collection, the parents were instructed to follow the specific procedures such as using dim lighting; restricted snack intake; avoiding chocolate and bananas; no eating 30 min prior to collection; avoiding caffeine, alcohol, and smoking; rinsing the subjects' mouth with water after eating or drinking; and not taking non-steroidal anti-inflammatory medications such as ibuprofen. The samples were kept frozen after collection, initially at home at −20°C and after in the laboratory at −80°C.

The melatonin samples were analyzed by using a salivary melatonin enzyme immunoassay kit (Salimetrics, PA, USA). The first non-zero standard of this assay was 0.78 pg/ml. Intra-assay coefficients of variation for low, medium, and high levels of salivary melatonin were 7.4, 3.3, and 3.9%, respectively. The inter-assay coefficients of variation for low, medium, and high levels were 15.6, 5.6, and 4.6%, respectively.

The time of DLMO was defined as the time at which evening salivary melatonin concentrations increased and remained above a 4 pg/ml threshold, using a linear interpolation between successive samples. In order to obtain a greater yield in melatonin analysis and given the difficulties involved in obtaining the samples (children had to spit in a tube five times and obtain enough quantity), melatonin samples are also described as the DLMO pattern using the following different descriptions (28):

° Normal: if concentration reaches the 4 pg/ml threshold during the recorded time and shows an upward trend.
° Advanced: if concentration reaches the 4 pg/ml threshold before the recorded time and shows an upward trend.
° Delayed: concentration does not reach the 4 pg/ml threshold during the recorded time.
° Irregular: concentration does not follow an identifiable trend.

Calculation of DLMO time was not possible in those cases that showed an advanced pattern in which concentration had already reached the 4 pg/ml threshold, prior to the scheduled time, and in some cases with an irregular trend in which no clear pattern of secretion was identified.

### Statistical Analyses

Descriptive statistics were used to analyze all major variables. The Shapiro–Wilk normality test was used as the basis for the selection of parametric or non-parametric statistical tests. Continuous variables were presented as mean and standard deviation (SD) or median and interquartile range (IQR, P25, P75). Categorical variables were expressed as percentages. A *t*-test for independent samples or a Mann–Whitney U test was used to assess differences between controls and participants with ASD. Subgroup analysis by age and the pubertal stage was performed for each variable under study. Correlations (Pearson or Spearman) were determined to measure the association between melatonin and quantitative variables such as age, BMI, and sleep and circadian parameters. The p-values of < 0.05 were considered statistically significant. Analyses were performed using IBM SPSS version 25 (New York, NY).

## Results

### Participants

In total, 56 autistic children and adolescents and 29 controls were initially enrolled in the study; of these, 15 autistic children and five controls were not included in the final sample due to inadequate saliva collection. Of note, there were no significant differences between the ASD and control groups as regards the number of participants lost due to problems with saliva collection (p = 0.79). Although, initially, both groups were matched in terms of age, sex, pubertal stage, and BMI, given the difficulties with saliva collection, the final sample was slightly different in terms of age and pubertal stage, with a wider range of age and individuals with Tanner stage V, within the autistic group. In order to equate, again, both groups, four individuals with ASD pertaining to Tanner V were finally not included in the sample. Characteristics of the study population are listed in Table 1.

**Table 1:** Characteristics of the sample.

	**ASD (*n* = 37)**	**TD controls** **(*n* = 24)**	***p***
Age (years ± SD) Range	9.4 ± 2.6 5–15	8.42 ± 2.4 5–13	NS
Sex (Male, Female)	Male 34 Female 3	Male 18 Female 6	NS
BMI (kg/m^2^)	18.6 ± 3.7	17.3 ± 2.4	NS
Pubertal stage (Tanner) (*n*/ %)	I = 24 II = 8 III = 4 IV = 1	I = 14 II = 6 III = 3 IV = 1	NS

*ASD, autism spectrum disorder; TD, typically developing*.

### Melatonin in Saliva

**Figure 1:**
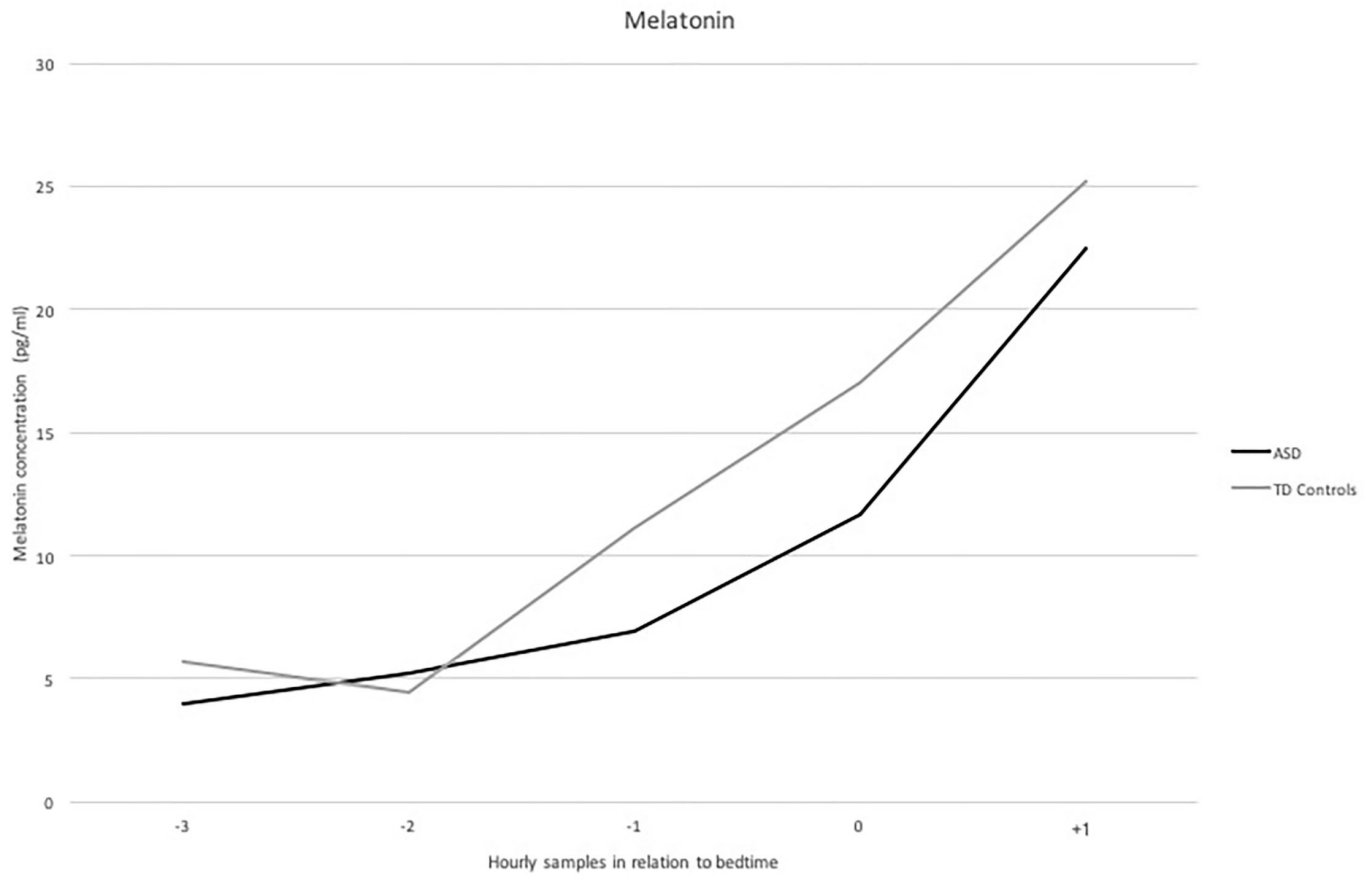
Levels of melatonin (pg/ml) in saliva (*Y* axis) for the period studied, i.e., −3, −2 and a−1 hour before bedtime, at bedtime and +1 hour after bedtime (X axis) in the ASD and control group.

Figure 1 displays concentrations of melatonin in saliva for the recorded period, showing how melatonin secretion increases more sharply in TD controls than among individuals in the ASD group. However, when analyzing differences between the different measurements (3, 2, and 1 h before bedtime; at bedtime; and 1 h after bedtime) in both groups, significance was not achieved.

The peak melatonin level in saliva was 22.09 (16.5–42.54) pg/ml in the ASD group (*n* = 35) and 31.46 (18.89–59.08) pg/ml among controls (*n* = 24), indicating a nonsignificant difference (*p* = 0.11). The time of peak melatonin concentration was 22.9 ± 1.4 h in the ASD group (*n* = 35) and 22.8 ± 1.1 h in the control group (*n* = 24), which also failed to reach statistical significance.

We determined DLMO in 18 autistic individuals and nine TD children and adolescents. The average DLMO was significantly later in ASD children than in the control group [21.58 ± 1.40 h vs. 20.41 ± 0.87 h (*P* < 0.05)]. No differences in terms of age, BMI, sex, or Tanner stage were detected for this subgroup of the sample.

The percentage of children and adolescents exhibiting each pattern of DLMO is shown in Table 2. Although the advanced profile was more frequent in the control group and the irregular pattern was the most commonly seen in the ASD group, statistical significance was not reached (*p* = 0.15 and *p* = 0.14). No participant in either groups presented the delayed subtype; although DLMO was later in autistic children, melatonin concentration reached the 4 pg/ml threshold during the recorded time in both groups. No differences were found when adjusting for pubertal stage or age.

**Table 2:** Patterns of DLMO in children with ASD and the control group and comparative analysis between groups.

	**ASD (*n* = 37%)**	**TD Controls (*n* = 24%)**	***p*** **-value**
Normal	13 (35%)	8 (33.3%)	NS
Advanced	10 (27%)	11 (45.8%)	NS
Delayed	0	0	NS
Irregular	14 (37%)	5 (20.8%)	NS

*ASD, autism spectrum disorder; TD, typically developing*.

### Melatonin and Age

The peak concentration of melatonin did not change with age in TD controls, although it did within the ASD group (>9 years: 22.05 ± 14.20, ≤9 years: 48.20 ± 45.23, *p* = 0.006). Therefore, a statistically significant negative correlation between the maximum concentration of melatonin in saliva and age was observed only in the ASD group (*r* = −0.42, *p* = 0.01). Furthermore, when we subdivided the study population into those older and younger than 9 years, we observed significant differences in the peak level of melatonin in saliva among those older than 9 years (ASD 28.29 ± 33.72 vs controls 42.62 ± 41.47, *p* = 0.04) but not in younger patients (ASD 47.13 ± 36.09 vs controls 43.48 ± 31.16, *p* = 0.87).

### Melatonin, Pubertal Stage, and BMI

After the pubertal stage was evaluated, significant differences in melatonin peak levels were found only in subjects with Tanner III (ASD 21.26 ± 4.20, TD controls 34.00 ± 4.04, *p* = 0.03). Differences between the ASD group and TD controls regarding melatonin peaks were found neither within Tanner I individuals (ASD 42.97 ± 40.95, TD controls 42.10 ± 28.11, *p* = 0.61) nor within Tanner stage II (ASD 19.26 ± 9.30, TD controls 53.35 ± 56.75, *p* = 0.19). BMI was not associated with peak melatonin level, peak time, or DLMO time in either groups.

### Sleep, Circadian Parameters, and Melatonin

Specific data on individuals included in this study with regard to sleep parameters are displayed in Table 3. Circadian parameters can be found in Supplemental Tables 2, 3; both sleep and circadian results were reported in our previous study (29). We observed significant differences between ASD and control groups in sleep and circadian parameters. The ASD group showed significantly longer sleep latencies and lower total sleep time and sleep efficiency (*p* < 0.01, *p* < 0.01, and *p* < 0.01, respectively). The stability of rhythms concerning temperature, motor activity, sleep, and light intensity was also lower in the ASD group.

**Table 3:** Description of sleep parameters (median and standard deviation) and a comparison analysis between the ASD and control groups.

	**ASD (*n* = 37)**	**Controls (*n* = 24)**	***P*** **-value**
Bedtime (h)	23.14 ± 0.91	22.61 ± 0.51	< 0.05
Sleep onset time (h)	23.49 ± 1.04	22.81 ± 0.56	< 0.01
Wake time (h)	8.03 ± 0.91	7.91 ± 0.50	NS
Total sleep time (min)	510.88 ± 46.90	546.63 ± 26.9	< 0.01
Sleep onset latency (SoL)	21.16 ± 15.58	11.60 ± 7.8	< 0.01
Sleep efficiency (%)	89.36 ± 4.48	92.30 ± 1.90	< 0.01
Awakenings (n/h)	3.75 ± 1.03	3.91 ± 0.6	NS
Total light during sleep, Me (lux)	0.02 ±1.22	0.04 ± 0.11	NS
Total light 2 h before sleep (lux)	13.22 ± 9.73	22.44 ± 13.39	< 0.01
Blue light 2 h before sleep (lux)	3.54 ± 3.28	5.66 ± 4.13	< 0.01
Exposure to outdoor light (hours)	0.78 ± 0.60	1.02 ± 0.53	< 0.05
Total light 2 h after waking (lux)	145.35 ± 327.16	182.79 ± 225.60	< 0.05
Blue light 2 h after waking (lux)	51.28 ± 127.41	60.76 ± 85.37	< 0.05
Time in movement 2 h before sleep (seconds)	12.88 ± 2.96	13.17 ±2.49	NS
Time in movement 2 h after waking (seconds)	13.94 ± 2.76	16.51 ± 2.96	< 0.05

In both groups, individuals with a later DLMO and melatonin peak time were more likely to have later bedtimes (ASD *r* = 0.53, p=0.02, TD controls *r* = 0.89, *p* = 0.001 and ASD *r* = 0.39, *p* = 0.02, TD controls *r* = 0.60, *p* = 0.002, respectively) and sleep onset times (ASD *r* = 0.59, p0.01, TD controls: *r* = 0.84, *p* = 0.004 and ASD *r* = 0.35, *p* = 0.04, TD controls *r* = 0.60, *p* = 0.001, respectively) (see Table 4). DLMO and melatonin peak time were also correlated with wake times only for the control group (*r* = 0.70, *p* = 0.03 and *r* = 0.50, *p* = 0.01). Prolonged SOL and an increased number of awakenings were associated with later DLMO in the ASD group (*r* = 0.49, *p* = 0.03; *r* = 0.55, *p* = 0.01, respectively). These correlations were not found in the control group (see Table 4).

**Table 4:** Correlations among DLMO, melatonin peak, melatonin peak time, and sleep parameters.

	**DLMO**		**Melatonin peak**		**Melatonin peak time**	
	**ASD** **(*n* = 18)**	**TD controls (*n* = 9)**	**ASD (*n* = 35)**	**TD controls** **(*n* = 24)**	**ASD** **(*n* = 35)**	**TD controls** **(*n* = 24)**
Total sleep time	*r* = −0.32, *p =* 0.18	*r* = −0.24, *p =* 0.51	*r* = 0.32, *p =* 0.06	*r* = 0.10, *p =* 0.63	*r* = −0.10, *p =* 0.54	*r* = −0.12, *p =* 0.56
Sleep onset latency	*r* = 0.49, *p =* 0.03	*r* = −0.50, *p =* 0.16	*r* = −0.36, *p =* 0.03	*r* = 0.13, *p =* 0.53	*r* = −0.08, *p =* 0.63	*r* = 0.17, *p =* 0.40
Awakenings	*r* = 0.55, *p =* 0.01	*r* = 0.25, *p =* 0.51	*r* = −0.10, *p =* 0.56	*r* = 0.22, *p =* 0.29	*r* = 0.09, *p =* 0.60	*r* = 0.00, *p =* 0.97
Sleep efficiency	*r* = −0.45 *p =* 0.05	*r* = 0.04, *p =* 0.92	*r* = 0.30, *p =* 0.07	*r* = −0.23, *p =* 0.26	*r* = −0.14, *p =* 0.41	*r* = −0.13, *p =* 0.53
Wake after sleep onset	*r* = 0.31, *p =* 0.20	*r* = 0.08, *p =* 0.83	*r* = 0.10, *p =* 0.55	*r* = 0.27, *p =* 0.18	*r* = 0.17, *p =* 0.33	*r* = −0.09, *p =* 0.67
Bedtime	*r* = 0.53, p = 0.02	*r* = 0.89, *p =* 0.001	*r* = −0.89, *p =* 0.61	*r* = 0.27, *p =* 0.18	*r* = 0.39, *p =* 0.02	*r* = 0.60, *p =* 0.002
Sleep onset time	*r* = 0.59, *p =* 0.01	*r* = 0.84, *p =* 0.004	*r* = −0.21, *p =* 0.23	*r* = 0.26, *p =* 0.21	*r* = 0.35, *p =* 0.04	*r* = 0.60, *p =* 0.001
Wake time	*r* = 0.34, *p =* 0.16	*r* = 0.70, *p =* 0.03	*r* = 0.15, *p =* 0.37	*r* = 0.41, *p =* 0.05	*r* = 0.15, *p =* 0.36	*r* = 0.50, *p =* 0.01
Total light during sleep	*r* = 0.43, *p =* 0.07	*r* = −0.57, *p =* 0.10	*r* = 0.06, *p =* 0.72	*r* = −0.11, *p =* 0.58	*r* = 0.09, *p =* 0.60	*r* = −0.13, *p =* 0.51
Total light 2 h before sleep	*r* = −0.14, r = 0.58	*r* = 0.09, *p =* 0.81	*r* = 0.10, *p =* 0.54	*r* = −0.33, *p =* 0.10	*r* = 0.07, *p =* 0.68	*r* = −0.14, *p =* 0.51
Exposure to outdoors lights	*r* = −0.25, *p =* 0.30	*r* = 0.34, *p =* 0.36	*r* = 0.13, *p =* 0.45	*r* = 0.17, *p =* 0.41	*r* = −0.25, *p =* 0.14	*r* = 0.03, *p =* 0.85
Total light 2 h after waking	*r* = −0.35, *p =* 0.14	*r* = 0.35, *p =* 0.34	*r* = 0.01, *p =* 0.94	*r* = 0.56, *p =* 0.004	*r* = −0.29, *p =* 0.09	*r* = 0.07, *p =* 0.73

When considering the different patterns of DLMO, only those participants in the ASD subgroup exhibiting the advanced pattern had shorter sleep onset latency than those with a normal or irregular pattern (13.56 ± 8.7 min vs. 24.22 ± 16.74 min, *p* = 0.02). In addition, in the autistic group, melatonin peak levels were correlated with sleep onset latency (*r* = −0.36, *p* = 0.03), indicating that higher levels of melatonin are related to shorter sleep latency. Total light 2 h after waking was related to melatonin peak levels in the TD control group (*r* = 0.56, *p* = 0.004).

Regarding temperature (see Table 5), DLMO time and peak melatonin time were associated with VM5 of temperature in TD controls, which points to the sleep midpoint measured by temperature (*r* = 0.81, *p* = 0.07 and *r* = 0.42, *p* = 0.03, respectively), indicating that a later DLMO and later peak melatonin time are related to higher values of temperature during sleep, and in the ASD group, DLMO showed a statistical correlation with VL10 time (daytime midpoint *r* = 0.63, *p* = 0.004), indicating that later VL10 time is related to later DLMO time. Melatonin peak time also showed a statistical correlation with VL10 time in both groups (ASD group: *r* = 0.40, *p* = 0.01, control group: *r* = 0.44, *p* = 0.02, indicating that later VL10 time is related to later melatonin peak time). Focusing on temperature data from 5:00 p.m. to 9:00 p.m., in the subgroup of TD controls with an irregular melatonin pattern, the mean temperature in this range of hours was higher than that for participants with a non-irregular pattern (irregular DLMO 33.76 ± 4.14, non-irregular DLMO 29.96 ± 2.17, *p* = 0.03). This finding was not observed in the ASD group.

**Table 5:** Melatonin and circadian parameters (temperature and TAP).

**Wrist temperature**							**TAP**						
	**DLMO time (h)**		**Melatonin peak (pg/ml)**		**Melatonin peak time (h)**			**DLMO time (h)**		**Melatonin peak (pg/ml)**		**Melatonin peak time (h)**	
	**ASD** **(*****n*** **= 18)**	**Control** **(*****n*** **= 9)**	**ASD** **(*****n*** **= 35)**	**Control** **(*****n*** **= 24)**	**ASD** **(*****n*** **= 35)**	**Control** **(*****n*** **= 24)**		**ASD** **(*****n*** **= 18)**	**Control** **(*****n*** **= 9)**	**ASD** **(*****n*** **= 35)**	**Control** **(*****n*** **= 24)**	**ASD** **(*****n*** **= 35)**	**Control** **(*****n*** **= 24)**
Mean	*r =* 0.25, *p =* 0.31	*r =* 0.24, *p =* 0.52	*r =* −0.44, *p =* 0.80	*r =* −0.10, *p =* 0.63	*r =* 0.17, *p =* 0.33	*r =* 0.29, *p =* 0.15							
IS	*r =* −0.40, *p =* 0.87	*r =* 0.25, *p =* 0.51	*r =* 0.18, *p =* 0.28	*r =* −0.03, *p =* 0.87	*r =* 0.25, *p =* 0.14	*r =* 0.15, *p =* 0.47	IS	*r =* −0.07, *p =* 0.97	*r =* 0.28, *p =* 0.46	*r =* 0.26, *p =* 0.12	*r =* −0.13, *p =* 0.51	*r =* 0.03, *p =* 0.83	*r =* 0.37, *p =* 0.07
IV	*r =* 0.36, *p =* 0.13	*r =* 0.19, *p =* 0.61	*r =* −0.24, *p =* 0.16	*r =* −0.33, *p =* 0.10	*r =* 0.12, *p =* 0.47	*r =* 0.30, *p =* 0.15	IV	*r =* 0.37, *p =* 0.12	*r =* 0.49, *p =* 0.17	*r =* −0.51, *p =* 0.002	*r =* −0.37, *p =* 0.07	*r =* 0.06, *p =* 0.71	*r =* 0.36, *p =* 0.07
NRA	*r =* −0.12, *p =* 0.61	*r =* −0.50, *p =* 0.89	*r =* 0.23, *p =* 0.18	*r =* 0.07, *p =* 0.73	*r =* 0.05, *p =* 0.74	*r =* 0.34, *p =* 0.09	NRA	*r =* −0.09, *p =* 0.70	*r =* 0.25, *p =* 0.51	*r =* 0.33, *p =* 0.05	*r =* −0.10, *p =* 0.63	*r =* −0.82, *p =* 0.64	*r =* 0.30, *p =* 0.14
M5	*r =* 0.16, *p =* 0.50	*r =* 0.51, *p =* 0.15	*r =* 0.08, *p =* 0.62	*r =* −0.17, *p =* 0.42	*r =* 0.03, *p =* 0.86	*r =* 0.31, *p =* 0.13	L5	*r =* 0.21, *p =* 0.39	*r =* 0.27, *p =* 0.47	*r =* −0.15, *p =* 0.38	*r =* −0.05, *p =* 0.78	*r =* −0.55, *p =* 0.75	*r =* 0.37, *p =* 0.06
VM5	*r =* 0.31, *p =* 0.19	*r =* 0.81, *p =* 0.07	*r =* 0.13, *p =* 0.46	*r =* −0.17, *p =* 0.40	*r =* 0.21, *p =* 0.21	*r =* 0.42, *p =* 0.03	VL5	*r =* −0.14, *p =* 0.55	*r =* −0.44, *p =* 0.23	*r =* −0.14, *p =* 0.42	*r =* 0.22, *p =* 0.78	*r =* −0.21, *p =* 0.23	*r =* −0.54, *p =* 0.005
L10	*r =* 0.63, *p =* 0.004	*r =* 0.43, *p =* 0.23	*r =* −0.03, *p =* 0.84	*r =* −0.37, *p =* 0.07	*r =* 0.40, *p =* 0.01	*r =* 0.44, *p =* 0.02	M10	*r =* 0.19, *p =* 0.43	*r =* −0.12, *p =* 0.74	*r =* 0.09, *p =* 0.60	*r =* −0.41, *p =* 0.04	*r =* −0.37, *p =* 0.03	*r =* 0.33, *p =* 0.10
VL10	*r =* 0.28, *p =* 0.25	*r =* 0.15, *p =* 0.70	*r =* −0.12, *p =* 0.47	*r =* −0.14, *p =* 0.50	*r =* 0.06, *p =* 0.72	*r =* −0.16, *p =* 0.44	VM10	*r =* −0.28, *p =* 0.25	*r =* 0.04, *p =* 0.91	*r =* 0.31, *p =* 0.07	*r =* 0.03, *p =* 0.87	*r =* −0.16, *p =* 0.33	*r =* −0.09, *p =* 0.67
CFI	*r =* −0.09, *p =* 0.72	*r =* 0.05, *p =* 0.89	*r =* 0.19, *p =* 0.27	*r =* 0.44, *p =* 0.83	*r =* 0.17, *p =* 0.32	*r =* 0.31, *p =* 0.13	CFI	*r =* 0.08, *p =* 0.73	*r =* 0.36, *p =* 0.33	*r =* 0.17, *p =* 0.32	*r =* −0.19, *p =* 0.37	*r =* 0.09, *p =* 0.58	*r =* 0.50, *p =* 0.01

*CFI, circadian function index; IS, inter-daily stability; IV, intradaily variability; NRA, normalized relative amplitude; M5, average measured for the 5 consecutive hours with the maximum values; L10, average measured for the 10 consecutive hours with the minimum values; L5, average measured for the 5 consecutive hours of minimum values; M10, average measured for the 10 consecutive hours of maximum values; TAP, integrated variable known as thermometry, actimetry, and body position*.

Concerning motor activity, we found a relation between melatonin peak time and DLMO time in the control group only (see Table 6). These results indicate that later melatonin peak time and DLMO time are related to lower motor activity and lower stability of its rhythms. Thus, higher values of melatonin peak are correlated with lower motor activity during sleep time (*r* = −0.44, *p* = 0.02). The same results were not found in the ASD group; however, when using the integrated variable “TAP” (thermometry, actimetry, body position), a correlation was detected between the melatonin peak level and intradaily variability (IV, *r* = −0.51, *p* = 0.002), indicating that higher levels of melatonin at night are associated with lower intradaily variability, which was not seen in the control group (see Table 5).

**Table 6:** Melatonin and motor activity parameters.

	**TD controls**		**ASD**	
	**Melatonin peak time (h)**	**DLMO time (h)**	**Melatonin peak time (h)**	**DLMO time (h)**
Mean motor activity	*r = –*0.53, *p =* 0.007	*r = –*0.68, *p =* 0.04	NS	NS
IS motor activity	*r = –*0.67, *p =* 0.000	NS	NS	NS
NRA motor activity	*r = –*0.55, *p =* 0.005	*r = –*0.75, *p =* 0.02	NS	NS
VM10 motor activity	*r = –*0.56, p = 0.004	*r =* 0.75, *p =* 0.02	NS	NS
CFI motor activity	*r = –*0.58, *p =* 0.003	NS	NS	NS
Mean time in movement	*r = –*0.61, *p =* 0.001	*r = –*0.73, *p =* 0.02	NS	NS
IS time in movement	*r = –*0.63, *p =* 0.001	*r = –*0.76, *p =* 0.01	NS	NS
NRA time in movement	*r = –*0.59, *p =* 0.002	*r =* 0.78, *p =* 0.01	NS	NS
VM10 time in movement	*r = –*0.59, *p =* 0.002	*r =* 0.78, *p =* 0.01	NS	NS
CFI time in movement	*r = –*0.60, *p =* 0.002	*r =* 0.77, *p =* 0.01	NS	NS

*ASD, autism spectrum disorder; TD, typically developing; IS, inter-daily stability; NRA, normalized relative amplitude; VM10, average measured for the 10 consecutive hours of maximum values; CFI, circadian function index*.

## Discussion

The principal aim of this study was to evaluate melatonin rhythm and its relationship with sleep and circadian parameters in drug-naive autistic children and adolescents. To our knowledge, this is the first study to include both sleep and circadian variables together with melatonin in an autistic pediatric population and the largest controlled study analyzing these data.

The main finding is that DLMO differed between participants with ASD and the control group, occurring later in the autistic group. Previous studies of individuals with ASD did not find differences in the timing of DLMO between ASD and TD controls (22, 24), although Baker et al. did find a greater variability in that timing in ASD individuals than in the control group (24). It should be noted that these studies only included adults and adolescents, and with respect to Goldman et al. (22), it is not clear that they excluded individuals under pharmacologic treatment. Other previous studies on prepubertal autistic subjects are limited, with the exception of the study by Goldman et al. (21) which did not focus on DLMO. Goldman et al. included a subgroup of nine participants, and DLMO was available for six of them, although there was no control group for comparison. The authors found no differences in DLMO when compared to previous data (21). Compared with research including other neurodevelopmental disorders such as ADHD, in this population, later DLMO has also been described, especially in the subgroup of individuals with sleep onset insomnia (25–27). In addition, in ADHD, it has been described how DLMO is modified using low doses of melatonin (38). In our study, no significant differences were found in the DLMO pattern, although the advanced subtype was more common within the control group. This finding may be consistent with the fact that DLMO occurs earlier in this group and is more pronounced during the period recorded. The delayed subtype was not identified in any group, and no significant phase shifts were observed. This finding may be related to the median age of our sample and the fact that the onset of psychological disorders such as depression or anxiety and delayed sleep–wake phase disorders typically increases during adolescence (39, 40).

The peak time of endogenous melatonin was not different between groups included in this study. However, as concerns the peak level during the period studied, older ASD individuals showed lower values than age-matched TD controls, and the maximum concentration of melatonin was inversely associated with age. Melatonin concentration is known to decline during adolescence, mainly between 15 and 20 years of age or in Tanner stages III-V (41–43). Taking into account that the median age in our sample was <15–20 years and that TD controls did not show this drop, we believe the decline in melatonin concentration could occur earlier in ASD. Although one hypothesis for this decrease in melatonin involves the relationship with body size (9, 21), BMI was not different within either groups and BMI was not associated with the melatonin peak. Other studies in healthy adults have documented an association between weight and melatonin secretion, with lower weight associated with an increased amplitude of melatonin secretion (9). Notably, this issue is scarcely described in children, and the evidence base is even weaker for the ASD population (21).

Previous investigations in ASD have suggested a role played by abnormal melatonin production in the etiology of sleep problems. However, these studies are methodologically different and apply varying inclusion criteria, thus resulting in controversial data. Specifically, changes in melatonin production among pubertal subjects with ASD have pointed to increased nocturnal levels of melatonin compared to younger participants with ASD (17). We found notable differences between our study and that of Tordjman et al., and these differences complicate efforts to compare them directly. First, we collected data from saliva samples in order to determine DLMO, while the authors of the other articles draw on nocturnal 6-sulfametoxymelatonin levels in urine collected from 8:00 p.m. to 8:00 a.m. Although the other team of researchers calculated excretion over the 12 h of collection, they could not determine the rhythm of this secretion. By contrast, our study did not measure melatonin concentration during the rest of the night or day, and it may be possible to have initially elevated levels of melatonin, followed by lower levels, and thus, data from different studies would not be contradictory.

Due to the design of our study, our findings cannot be compared to other reports describing reduced amplitude of melatonin rhythms, including diminished nocturnal values, as well as melatonin rhythm abnormalities such as inverted circadian rhythms (15–17, 44–47). Other authors have suggested the possible influence of medication on melatonin levels as they found lower concentrations only in the subgroup of adults with ASD under medication for psychologic disorders (24); others, in contrast, have not found any differences (21). Given this variability, it would be of interest to conduct studies that assess melatonin levels for several days, during daytime and nighttime, and to simultaneously study the possible state of hyperserotonemic and normoserotonemic groups and the activity of the different enzymes involved in the process, which could also explain these differences (48, 49). The serotonergic system has been previously implicated in the pathogenesis of ASD. Serotonin can be measured in platelets, and previous studies have shown that approximately one-third of autistic people have hyperserotonemia and also that individuals with ASD can display platelet hyposerotonemia, with both states implicated in ASD symptoms (49). This points to a bidirectional dysregulation of serotonin in ASD, and since serotonin is the precursor of the hormone melatonin, this imbalance could be affecting melatonin secretion.

A relationship was found between melatonin and both sleep and circadian parameters. Participants with later DLMOs in both groups were more likely to have later bedtimes and sleep onset times, a finding consistent with that of previous research on TD individuals (50, 51). In the previous reports, DLMO was also related to wake times as in our TD control group but not in our subjects with ASD. It is difficult to explain the reason for this aspect. We hypothesize that it may be related to the irregular pattern of melatonin secretion that is more prevalent in the ASD group and also the different exposure to light throughout the day, with especially lower values of total and blue light in the ASD group in the morning (see Supplementary Table 3). In fact, we have described that, in TD controls, higher exposure to total light 2 h after waking is related to higher melatonin peak levels, which agrees with previous knowledge about melatonin secretion and light, as diurnal bright light has been suggested to increase melatonin secretion and to have a preventive impact on light-induced melatonin suppression at night (52).

Autistic children and adolescents went to bed later than TD controls, which is in agreement with the later DLMO found. Moreover, among these ASD participants with later bedtimes, latency and awakening were associated with this later DLMO. In addition, the ASD subgroup exhibiting an advanced pattern of DLMO also had shorter sleep latency than that with a normal or irregular pattern, and higher levels of melatonin were related to shorter sleep latency. It has been previously hypothesized that abnormal melatonin rhythms could be the cause of altered sleep parameters in ASD (20); our results mostly support this analysis, although it must be noted that the time of DLMO and not the level of melatonin peak was related to abnormal sleep parameters in our study. In the study by Baker et al. (24) the authors found a relation between sleep problems and melatonin levels, with increases in melatonin, prior to and after habitual sleep time, being associated with reduced sleep onset latency and decreased wake after sleep onset. To our knowledge, no other studies have analyzed this relationship.

As commented before, we found that circadian parameters were also related to melatonin. Some of these relationships have been found in the ASD group or in the control group only, though always independently. Once again, it is difficult to explain the reason for this. In our opinion, this could be related to the small study sample and also to differences in the pattern of melatonin between the two groups. To our knowledge, ours is the first study to describe a relationship between DLMO and circadian parameters in an autistic population. It is well-known that melatonin increases peripheral blood flow, thus lowering core temperature, which is associated with sleep onset (10, 53, 54). In this study, DLMO seems to be related to daytime and nighttime wrist temperature values, with later DLMO and later peak melatonin time in those with the delayed daytime midpoint. Due to the lack of studies on this issue in ASD, it is not possible to compare. Once again, it would be of interest to study melatonin levels for several days, during daytime and nighttime, and its relationship with temperature, in order to better define their association.

When analyzing motor activity, it showed a clear interaction with melatonin in TD controls as later melatonin peak time and DLMO time were related to lower motor activity and lower stability of its rhythm. The same has not been found in the ASD group, which could be explained by the overall lower motor activity in ASD subjects and also by a different intrinsic pattern of melatonin, which could be differently affected by exercise. In the ASD group, nonetheless, stability of temperature and motor activity were higher in those with increased nighttime levels of melatonin. Previous knowledge on the effect of physical exercise on endogenous melatonin production is controversial as it has been shown that melatonin levels can increase, decrease, or remain unaffected by exercise (55, 56). On the other hand, physical exercise has been suggested as a synchronizer of the circadian system, making it a potential treatment for circadian rhythm misalignment (55, 57, 58). As a result, exercise may be an accessible non-pharmacological intervention of special interest when treating ASD individuals. In any case, further large-scale studies focusing on circadian parameters and melatonin during the daytime and nighttime are needed.

Several limitations should be considered when interpreting this study. First, saliva collection was not available in some of the participants, and some data may be missing from children with more severe forms of ASD as collection could be more challenging for them. Second, we were unable to measure DLMO in all individuals as some cases showed advanced or irregular trends that preclude such measurement; as a result, we obtained a relatively small sample size. In addition, our study focused exclusively on the hours around sleep, and no data on melatonin are available for the rest of the day and night. Also, only one night has been evaluated here, and in order to better define the melatonin secretion patterns, it would be recommended that study should be carried out in different days of the week. Future studies should, therefore, extend melatonin measurements.

## Conclusion

Melatonin, which is related to sleep and circadian parameters, exhibits different patterns of secretion in ASD. In the ASD group, melatonin seems to decline earlier with age. Expanding the knowledge base on melatonin and its relation to temperature and motor activity could aid in establishing the causes of this intrinsic characteristic in ASD. The ACM device used here makes it possible to objectively study sleep and circadian data, and it can be used as a complementary tool when studying melatonin secretion. Finally, it would be of great interest to implement larger studies, multi-day studies, including objective sleep and circadian parameters, with the determination of hyperserotonemic and nor-moserotonemic groups.

## Data Availability Statement

The datasets presented in this study can be found in online repositories. The names of the repository and accession number can be found below: Open Science Framework (OSF), https://osf.io/jkb24/.

## Ethics Statement

The studies involving human participants were reviewed and approved by Fundación Jiménez Díaz review board (code: PIC018_18FJD, approval date: 3/13/2018. Written informed consent to participate in this study was provided by the participants' legal guardian/next of kin.

## Author Contributions

EM-C, TG-P, MM-A, and LS-G assisted in study conception and design. EM-C and MR-M contributed to data collection. EM-C, CV-V, IP-N, and CG performed data analysis. EM-C, TG-P, MM-A, and LS-G accomplished data interpretation and critical revision of the article. All authors contributed to the article and approved the submitted version.

## Funding

This work was supported by Fundación Familia Alonso (grant number: PIC006-18).

## Conflict of Interest

The authors declare that the research was conducted in the absence of any commercial or financial relationships that could be construed as a potential conflict of interest.

## Publisher's Note

All claims expressed in this article are solely those of the authors and do not necessarily represent those of their affiliated organizations, or those of the publisher, the editors and the reviewers. Any product that may be evaluated in this article, or claim that may be made by its manufacturer, is not guaranteed or endorsed by the publisher.

## References

[1] Geoffray MM Nicolas A Speranza M Georgieff N. Are circadian rhythms new pathways to understand Autism spectrum disorder? J Physiol Paris. (2016) 110:434–8. 10.1016/j.jphysparis.2017.06.002 28625682

[2] Goldman SE Surdyka K Cuevas R Adkins K Wang L Malow BA. Defining the sleep phenotype in children with autism. Dev Neuropsychol. (2009) 34:560–73. 10.1080/87565640903133509 20183719 PMC2946240

[3] Williams Buckley A Hirtz D Oskoui M Armstrong MJ Batra A Bridgemohan C. Practice guideline: treatment for insomnia and disrupted sleep behavior in children and adolescents with autism spectrum disorder: report of the guideline development, dissemination, and implementation subcommittee of the American academy of neurology. Neurology. (2020) 94:392–404. 10.1212/WNL.0000000000009033 32051244 PMC7238942

[4] Mazurek MO Petroski GF. Sleep problems in children with autism spectrum disorder: Examining the contributions of sensory over-responsivity and anxiety. Sleep Med. (2015) 16:270–9. 10.1016/j.sleep.2014.11.006 25600781

[5] Baglioni C Nanovska S Regen W Spiegelhalder K Feige B Nissen C. Sleep and mental disorders: a meta-analysis of polysomnographic research. Psychol Bull. (2016) 142:969–90. 10.1037/bul0000053.SLEEP 27416139 PMC5110386

[6] Becker SP. ADHD and sleep: recent advances and future directions. Curr Opin Psychol. (2020) 34:50–6. 10.1016/j.copsyc.2019.09.006 31629217 PMC7082190

[7] Reynolds AM Malow BA. Sleep and Autism Spectrum Disorders. Pediatr Clin North Am. (2011) 58:685–98. 10.1016/j.pcl.2011.03.009 21600349

[8] Tordjman S Anderson GM Bellissant E Botbol M Charbuy H Camus F. Day and nighttime excretion of 6-sulphatoxymelatonin in adolescents and young adults with autistic disorder. Psychoneuroendocrinology. (2012) 37:1990–7. 10.1016/j.psyneuen.2012.04.013 22613035

[9] Burgess HJ Fogg LF. Individual differences in the amount and timing of salivary melatonin secretion. PLoS ONE. (2008) 3:e3055. 10.1371/journal.pone.0003055 18725972 PMC2516604

[10] Van Someren EJW. More than a marker: Interaction between the circadian regulation of temperature and sleep, age-related changes, and treatment possibilities. Chronobiol Int. (2000) 4:1050. 10.1081/CBI-100101050 10841209

[11] Benloucif S Guico MJ Reid KJ Wolfe LF. L'Hermite-Balériaux M, Zee PC. Stability of melatonin and temperature as circadian phase markers and their relation to sleep times in humans. J Biol Rhythms. (2005) 20:178–88. 10.1177/0748730404273983 15834114

[12] Zisapel N. New perspectives on the role of melatonin in human sleep, circadian rhythms and their regulation. Br J Pharmacol. (2018) 175:3190–9. 10.1111/bph.14116 29318587 PMC6057895

[13] Pandi-Perumal SR Smits M Spence W Srinivasan V Cardinali DP Lowe AD. Dim light melatonin onset (DLMO): A tool for the analysis of circadian phase in human sleep and chronobiological disorders. Prog Neuro-Psychopharmacology Biol Psychiatry. (2007) 31:1–11. 10.1016/j.pnpbp.2006.06.020 16884842

[14] Mandrell BN Avent Y Walker B Loew M Tynes BL Crabtree VML. In-home salivary melatonin collection: Methodology for children and adolescents. Dev Psychobiol. (2018) 60:118–22. 10.1002/dev.21584 29152732 PMC5748004

[15] Kulman G Lissoni P Rovelli F Roselli MG Brivio F Sequeri P. Evidence of pineal endocrine hypofunction in autistic children. Neuroendocrinol Lett. (2000) 21:31–4. 11455326

[16] Nir I Meir D Zilber N Knobler H Hadjez J Lerner Y. Brief report: Circadian melatonin, thyroid-stimulating hormone, prolactin, and cortisol levels in serum of young adults with autism. J Autism Dev Disord. (1995) 25:641–54. 10.1007/BF02178193 8720032

[17] Tordjman S Anderson GM Pichard N Charbuy H Touitou Y. Nocturnal excretion of 6-sulphatoxymelatonin in children and adolescents with autistic disorder. Biol Psychiatry. (2005) 57:134–8. 10.1016/j.biopsych.2004.11.003 15652871

[18] Lalanne S Fougerou-Leurent C Anderson GM Schroder CM Nir T Chokron S. Molecular sciences melatonin: from pharmacokinetics to clinical use in autism spectrum disorder melatonin: from pharmacokinetics to clinical use in autism spectrum. Disord Int J Mol Sci. (2021) 22:1490. 10.3390/ijms22031490 33540815 PMC7867370

[19] Tordjman S Davlantis KS Georgieff N Geoffray M-M Speranza M Anderson GM. Autism as a disorder of biological and behavioral rhythms: toward new therapeutic perspectives. Front Pediatr. (2015) 3:1. 10.3389/fped.2015.00001 25756039 PMC4337381

[20] Glickman G. Circadian rhythms and sleep in children with autism. Neurosci Biobehav Rev. (2010) 34:755–68. 10.1016/j.neubiorev.2009.11.017 19963005

[21] Goldman SE Adkins KW Calcutt MW Carter MD Goodpaste RL Wang L. Melatonin in children with autism spectrum disorders: endogenous and pharmacokinetic profiles in relation to sleep. J Autism Dev Disord. (2014) 44:2525–35. 10.1007/s10803-014-2123-9 24752680 PMC4372534

[22] Goldman SE Alder ML Burgess HJ Corbett BA Hundley R Wofford D. Characterizing sleep in adolescents and adults with autism spectrum disorders. J Autism Dev Disord. (2017) 47:1682–95. 10.1007/s10803-017-3089-1 28286917 PMC5433911

[23] Braam W Keijzer H Struijker Boudier H Didden R Smits M Curfs L. CYP1A2 polymorphisms in slow melatonin meta-bolisers: a possible relationship with autism spectrum disorder? J Intellect Disabil Res. (2013) 57:993–1000. 10.1111/j.1365-2788.2012.01595.x 22823064

[24] Baker EK Richdale AL Hazi A Prendergast LA. Assessing the dim light melatonin onset in adults with autism spectrum disorder and no comorbid intellectual disability. J Autism Dev Disord. (2017) 47:2120–37. 10.1007/s10803-017-3122-4 28444476

[25] Snitselaar MA Smits MG van der Heijden KB Spijker J. Sleep and circadian rhythmicity in adult ADHD and the effect of stimulants: a review of the current literature. J Atten Disord. (2017) 21:14–26. 10.1177/1087054713479663 23509113

[26] Van Der Heijden KB Smits MG Van Someren EJW Gunning WB. Idiopathic chronic sleep onset insomnia in attention-deficit/hyperactivity disorder: a circadian rhythm sleep disorder. Chronobiol Int. (2005) 22:559–70. 10.1081/CBI-200062410 16076654

[27] Van Veen MM Kooij JJS Boonstra AM Gordijn MCM Van Someren EJW. Delayed circadian rhythm in adults with attention-deficit/hyperactivity disorder and chronic sleep-onset Insomnia. Biol Psychiatry. (2010) 67:1091–6. 10.1016/j.biopsych.2009.12.032 20163790

[28] Pitarch Castellano I. Ritmo Circadiano de secreción de Melatonina en niños con Trastorno por Déficit de Atención con Hiperactividad (TDAH). (2016). 229 p.

[29] Castellano IP. Ritmo Circadiano de secreción de Melatonina en niños con Trastorno por Déficit de Atención con Hiperactividad (TDAH). Moncada (Valencia): Universidad CEU Cardenal Herrera, Facultad de Ciencias de la Salud, Departamento de Ciencias Biomédicas (2016).

[30] Lord C Risi S Lambrecht L Cook EH Leventhal BL Dilavore PC. The autism diagnostic observation schedule-generic: a standard measure of social and communication deficits associated with the spectrum of autism. J Autism Dev Disord. (2000) 30:205–23. 10.1023/A:1005592401947 11055457

[31] Budimirovic DB Protic DD Delahunty CM Andrews HF Choo TH Xu Q. Sleep problems in fragile X syndrome: Cross-sectional analysis of a large clinic-based cohort. Am J Med Genet Part A. (2022) 188:1029–39. 10.1002/ajmg.a.62601 34889523 PMC11057226

[32] Van Der Heijden KB Smits MG Gunning WB. Sleep hygiene and actigraphically evaluated sleep characteristics in children with ADHD and chronic sleep onset insomnia. J Sleep Res. (2006) 15:55–62. 10.1111/j.1365-2869.2006.00491.x 16490003

[33] Marshall WA Tanner JM. Variations in pattern of pubertal changes in girls. Arch Dis Child. (1969) 44:291–303. 10.1136/adc.44.235.291 5785179 PMC2020314

[34] Marshall WA Tanner JM. Variations in the pattern of pubertal changes in boys. Arch Dis Child. (1970) 45:13–23. 10.1136/adc.45.239.13 5440182 PMC2020414

[35] Ortiz-Tudela E Martinez-Nicolas A Albares J Segarra F Campos M Estivill E. Ambulatory circadian monitoring (ACM) based on thermometry, motor activity and body position (TAP): a comparison with polysomnography. Physiol Behav. (2014) 126:30–8. 10.1016/j.physbeh.2013.12.009 24398067

[36] Van Someren EJW Riemersma-Van RF Lek D. Live to the rhythm, slave to the rhythm. Sleep Med Rev. (2007) 11:465–84. 10.1016/j.smrv.2007.07.003 18021942

[37] Ortiz-Tudela E Martinez-Nicolas A Campos M Rol M Madrid MA. A new integrated variable based on thermometry, actimetry and body position (TAP) to evaluate circadian system status in humans. PLoS Comput Biol. (2010) 6:e1000996. 10.1371/journal.pcbi.1000996 21085644 PMC2978699

[38] van Andel E Bijlenga D Vogel SWN Beekman ATF Kooij JJS. Effects of chronotherapy on circadian rhythm and ADHD symptoms in adults with attention-deficit/hyperactivity disorder and delayed sleep phase syndrome: a randomized clinical trial. Chronobiol Int. (2021) 38:260–9. 10.1080/07420528.2020.1835943 33121289

[39] American Academy of Sleep. International Classification of Sleep Disorders (3rd edn.). (2014).

[40] Gotham K Brunwasser S Lord C. Depressive and anxiety symptom trajectories from school-age through young adulthood in samples with autism spectrum disorder and developmental delay. J Am Acad Child Adolesc Psychiatry. (2015) 54:369–76. 10.1016/j.jaac.2015.02.005.Depressive 25901773 PMC4407021

[41] Bruni O Alonso-Alconada D Besag F Biran V Braam W Cortese S. Current role of melatonin in pediatric neurology: clinical recommendations. Eur J Paediatr Neurol. (2015) 19:122–33. 10.1016/j.ejpn.2014.12.007 25553845

[42] Cavallo A Ritschel WA. Pharmacokinetics of melatonin in human sexual maturation. J Clin Endocrinol Metab. (1996) 81:1882–6. 10.1210/jc.81.5.1882 8626852

[43] Salti R Galluzzi F Bindi G Perfetto F Tarquini R Halberg F. Nocturnal melatonin patterns in children. J Clin Endocrinol Metab. (2000) 85:2137–44. 10.1210/jcem.85.6.6656 10852442

[44] Abdulamir HA Abdul-Rasheed OF Abdulghani EA. Low oxytocin and melatonin levels and their possible role in the diagnosis and prognosis in Iraqi autistic children. Saudi Med J. (2016) 37:29–36. 10.15537/smj.2016.1.13183 26739971 PMC4724675

[45] Babinska K Siklenkova L Stebelova K Waczulikova I Celusakova H Vidosovicova M. Urinary levels of 6-sulphatoxymelatonin and their associations with sleep disorders and behavioural impairments in children with autism spectrum disorder. Bratisl Med J. (2019) 120:849–55. 10.4149/BLL 31747766

[46] Liang Y Xiao Z Ke X Yao P Chen Y Lin L. Urinary Metabonomic profiling discriminates between children with autism and their healthy siblings. Med Sci Monit. (2020) 26:1–8. 10.12659/MSM.926634 33237888 PMC7702663

[47] Melke J Goubran Botros H Chaste P Betancur C Nygren G Anckarsäter H. Abnormal melatonin synthesis in autism spectrum disorders. Mol Psychiatry. (2008) 13:90–8. 10.1038/sj.mp.4002016 17505466 PMC2199264

[48] Mulder EJ Anderson GM Kemperman RFJ Oosterloo-Duinkerken A Minderaa RB Kema IP. Urinary excretion of 5-hydroxyindoleacetic acid, serotonin and 6-sulphatoxymelatonin in normoserotonemic and hyperserotonemic autistic individuals. Neuropsychobiology. (2009) 61:27–32. 10.1159/000258640 19923863

[49] Garbarino VR Gilman TL Daws LC Gould GG. Extreme enhancement or depletion of serotonin transporter function and serotonin availability in autism spectrum disorder. Pharmacol Res. (2019) 140:85–99. 10.1016/j.phrs.2018.07.010 30009933 PMC6345621

[50] Lebourgeois MK Carskadon MA Akacem LD Simpkin CT Wright KP Achermann P. Circadian phase and its relationship to nighttime sleep in toddlers. J Biol Rhythm. (2013) 28:322–31. 10.1177/0748730413506543.Circadian 24132058 PMC3925345

[51] Sadeh A. Sleep and melatonin in infants: a preliminary study. Sleep. (1997) 20:185–91. 10.1093/sleep/20.3.185 9178914

[52] Kozaki T Kubokawa A Taketomi R Hatae K. Light-induced melatonin suppression at night after exposure to different wavelength composition of morning light. Neurosci Lett. (2016) 616:1–4. 10.1016/j.neulet.2015.12.063 26777427

[53] Bonmati-Carrion MA Middleton B Revell V Skene DJ Rol MA Madrid JA. Circadian phase asessment by ambulatory monitoring in humans: correlation with Dim Light Melatonin Onset. Chronobiol Int. (2014) 31:37–51. 10.3109/07420528.2013.820740 24164100

[54] Sarabia JA Rol MA Mendiola P Madrid JA. Circadian rhythm of wrist temperature in normal-living subjects. A candidate of new index of the circadian system. Physiol Behav. (2008) 95:570–80. 10.1016/j.physbeh.2008.08.005 18761026

[55] Escames G Ozturk G Baño-Otálora B Pozo MJ Madrid JA Reiter RJ. Exercise and melatonin in humans: reciprocal benefits. J Pineal Res. (2012) 52:1–11. 10.1111/j.1600-079X.2011.00924.x 21848991

[56] Stacchiotti A Favero G Rodella LF. Impact of melatonin on skeletal muscle and exercise. Cells. (2020) 9:1–23. 10.3390/cells9020288 31991655 PMC7072499

[57] Brand S Jossen S Holsboer-Trachsler E Pühse U Gerber M. Impact of aerobic exercise on sleep and motor skills in children with autism spectrum disorders – A pilot study. Neuropsychiatr Dis Treat. (2015) 11:1911–20. 10.2147/NDT.S85650 26346856 PMC4531010

[58] Tse ACY Lee PH Zhang J Chan RCY Ho AWY Lai EWH. Effects of exercise on sleep, melatonin level, and behavioral functioning in children with autism. Autism. (2022) 8:2952. 10.1177/13623613211062952 35083939

