# Supplementary material for: Melatonin Rhythm and Its Relation to Sleep and Circadian Parameters in Children and Adolescents With Autism Spectrum Disorder
Source: Front Neurol. 2022 Jun 14;13:813692. doi: 10.3389/fneur.2022.813692 (PMC9237227; doi:10.3389/fneur.2022.813692)
Supplement: Supplementary file 1 [file Data_Sheet_1.pdf]

Supplementary material:

Table 1: Major studies of melatonin secretion in ASD

References / Country	ASD study group	ID/DD included (Yes, no, not reported)	Sample sizes (ASD/ TDC)	Variable measured	Sample timing	24-hour rhythm determined (yes/no)	Mean age (yr, SD)	Medication	Main outcomes (ASD group)
(Tordjman et al., 2012) France	ASD-cognitively impaired	Yes	43/26	Day and nighttime urinary excretion of 6-sulphatoxymelatonin	Urine sampled across 24-hours	Yes	18.6 (0.5)	Unmedicated for at least one month before urine collection	Low 6-SM excretion both in daytime and at nighttime. Smaller intraindividual nighttime-daytime differences in ASD patients Nocturnal excretion of 6-SM negatively correlated with ASD severity.
(Tordjman et al., 2005) France	ASD-cognitively impaired Comorbid epilepsy included	Yes	49/88	nighttime urinary excretion of 6-sulphatoxymelatonin	Urine sampled across 12-hours	No	11.5 (4.5)	Yes	Lower nocturnal 6-SM excretion Nocturnal excretion of 6-SM negatively correlated with ASD severity
(Melke et al., 2008) France, Sweden, Norway, Italy, Belgium, Austria, USA	ASD with IQ<70	Not reported	43/48	Melatonin concentration in plasma. ASMT activity	Fixed (between 09:00 and 11:00h). One family plasma sampled across 22 hours	No	33 (6)	Not described	Lower plasma melatonin concentration in patients with ASD Decreased ASMT activity
(Nir et al., 1995) Israel	ASD Comorbid epilepsy included "Extreme autism disorder"	Yes (described as Speech disability)	10/5	Melatonin concentration in plasma.	Plasma sampled across 24-hours	Yes	16-30 years	Yes	Higher serum melatonin concentration during the day and lower during the night Smaller change in nychthemeral amplitude
(Kulman et al., 2000) Italy	"Classical infantile autism"	Not reported	14/20	Melatonin concentration in plasma.	Plasma sampled across 24-hours	Yes	Median age 7 years (range 5-10)	No	Lower mean concentrations of melatonin mainly during the dark phase of the day
(Mulder et al., 2009) The Netherlands	ASD Hyperserotonemic and normoserotonemic groups	Yes	20	Urinary excretion of 6-sulphatoxymelatonin	Urine sampled across 24-hours	Yes	15.3 (4.4)	No	Excretion of 6-SM decreased in the hyperserotonemic group Negative correlation of urinary excretion of 6-SM with platelet serotonin
(Maruani et al., 2019) France	ASD	Yes	81/48	Plasma melatonin level Pineal gland volume (PGV) MRI	Fixed (between 08:30 and 10:30h)	No	13.2 (8.8)	Not described	Morning plasma melatonin level was mainly associated with the study group and, to a lesser extent, but not significantly, with PGV. The effect of PGV on melatonin was weak. Low melatonin levels were detected in the probands and to a lesser extent in their relatives
(Babinska et al., 2019) Slovakia	ASD	Not reported	77/84	Morning and afternoon levels of 6-SM in urine	Fixed: first morning sample after wake up, and afternoon sample at lunch-time.	No	4.9 (range 2.6-14.9)	Not described	Nighttime melatonin levels found to be significantly lower in ASD with no differences in daytime levels. Lower nighttime increase in melatonin levels The difference in nighttime-daytime values correlated with some types of sleep disorders but not with ASD severity.
(Leu et al., 2011) USA	ASD	No	24/0	Nocturnal urine 6-SM rates and sleep architecture (PSG)	Fixed: first morning sample	No	5.7 (1.9)	No	Higher urinary 6-SM excretion rates were associated with increased N3 sleep, decreased N2 sleep and decreased daytime sleepiness.
(Baker et al., 2017) Australia	ASD	No	28/32	Saliva collection (evening and morning samples).	Relative to individual sleep onset	No	33.82 (6.99)	Yes	Mean melatonin levels were lower in adults with ASD medicated for comorbid diagnoses

									Greater increases in melatonin in the hour prior to sleep were associated with greater sleep efficiency in the ASD groups<br>The timing of DLMO did not differ<br>Advances and delays in melatonin rhythm were observed in individual profiles
(Abdulamir et al., 2016)<br><br>Iraq	ASD	Not reported	60/26	Melatonin and oxytocin concentration in plasma at nighttime	Fixed: 9:00h	No	7.28 (2.89)	Not described	Lower levels of oxytocin and melatonin in ASD<br>These parameters were associated with the severity of ASD.
(Liang et al., 2020)<br><br>China	ASD and their matched healthy siblings	Not reported	22/22	Morning first-pass urine samples	Fixed: first morning sample	No	7.6 (1.8)	Not described	Lower levels of melatonin in ASD
(da Silveira Cruz-Machado et al., 2021)<br>Brazil	ASD	Not reported	20/20	Daily urinary 6-SM excretion<br>Salivary levels of tumor necrosis factor and interleukin-6	Urine sampled across 24-hours	Yes	11 (4.9)	No	60% of ASD participants showed a significant increase in nocturnal 6-SM but this rise was significantly attenuated compared to controls.<br>40% of ASD individuals showed no significant increase in nocturnal 6-SM
(S. E. Goldman et al., 2017)<br>USA	ASD	Not reported	28/13	Salivary melatonin<br>Actigraphy	Relative to individual sleep onset	No	15.6 (2.8)	Yes (with the exception of melatonin and stimulants).	Dim light melatonin onset (DLMO) was not different between ASD and TD participants
(Suzanne E. Goldman et al., 2014)<br>USA	ASD	Not reported	9/0	Plasma melatonin level<br>PSG<br>Actigraphy	Relative to individual sleep onset	No	4.7 (1.5)	No	Maximal melatonin concentration and time to peak concentration were comparable to those published for typically developing children

Abbreviations: ASD: autism spectrum disorder; ASMT: acetylserotonin methyltransferase; ID/DD: Intellectual disability/developmental delay; 6-SM: 6-sulphatoxymelatonin; MRI: magnetic resonance imaging; PSG: polysomnography.

REFERENCES

Abdulamir, H. A., Abdul-Rasheed, O. F., & Abdulghani, E. A. (2016). Low oxytocin and melatonin levels and their possible role in the diagnosis and prognosis in Iraqi autistic children. *Saudi Medical Journal*, *37*(1), 29–36. https://doi.org/10.15537/smj.2016.1.13183

Babinska, K., Siklenkova, L., Stebelova, K., Waczulikova, I., Celusakova, H., Vidosovicova, M., Bartakovicova, K., Szapuova, Z., & P, K. (2019). Urinary levels of 6-sulphatoxymelatonin and their associations with sleep disorders and behavioural impairments in children with autism spectrum disorder. *Bratisl Med J*, *120*(11), 849–855. https://doi.org/10.4149/BLL

Baker, E. K., Richdale, A. L., Hazi, A., & Prendergast, L. A. (2017). Assessing the Dim Light Melatonin Onset in Adults with Autism Spectrum Disorder and No Comorbid Intellectual Disability. *Journal of Autism and Developmental Disorders*, *47*(7), 2120–2137. https://doi.org/10.1007/s10803-017-3122-4

da Silveira Cruz-Machado, S., Guissoni Campos, L. M., Fadini, C. C., Anderson, G., Markus, R. P., & Pinato, L. (2021). Disrupted nocturnal melatonin in autism: Association with tumor necrosis factor and sleep disturbances. *Journal of Pineal Research*, *70*(3), 1–11. https://doi.org/10.1111/jpi.12715

Goldman, S. E., Alder, M. L., Burgess, H. J., Corbett, B. A., Hundley, R., Wofford, D., Fawkes, D. B., Wang, L., Laudenslager, M. L., & Malow, B. A. (2017). Characterizing Sleep in Adolescents and Adults with Autism Spectrum Disorders. *Journal of Autism and Developmental Disorders*, *47*(6), 1682–1695. https://doi.org/10.1007/s10803-017-3089-1

Goldman, Suzanne E., Adkins, K. W., Calcutt, M. W., Carter, M. D., Goodpaste, R. L., Wang, L., Shi, Y., Burgess, H. J., Hachey, D. L., & Malow, B. A. (2014). Melatonin in Children with Autism Spectrum Disorders: Endogenous and Pharmacokinetic Profiles in Relation to Sleep. *Journal of Autism and Developmental Disorders*, *44*(10), 2525–2535. https://doi.org/10.1007/s10803-014-2123-9

Kulman, G., Lissoni, P., Rovelli, F., Roselli, M. G., Brivio, F., & Sequeri, P. (2000). Evidence of pineal endocrine hypofunction in autistic children. *Neuroendocrinology Letters*, *21*(1), 31–34.

Leu, R. M., Beyderman, L., Botzolakis, E. J., Surdyka, K., Wang, L., & Malow, B. A. (2011). Relation of melatonin to sleep architecture in children with autism. *Journal of Autism and Developmental Disorders*, *41*(4), 427–433. https://doi.org/10.1007/s10803-010-1072-1

Liang, Y., Xiao, Z., Ke, X., Yao, P., Chen, Y., Lin, L., & Lu, J. (2020). Urinary Metabonomic profiling discriminates between children with autism and their healthy siblings. *Medical Science Monitor*, *26*, 1–8. https://doi.org/10.12659/MSM.926634

Maruani, A., Dumas, G., Beggiato, A., Traut, N., Peyre, H., Cohen-Freoua, A., Amsellem, F., Elmaleh, M., Germanaud, D., Launay, J. M., Bourgeron, T., Toro, R., & Delorme, R. (2019). Morning plasma melatonin differences in autism: Beyond the impact of pineal gland volume. *Frontiers in Psychiatry*, *10*(11), 1–9. https://doi.org/10.3389/fpsyt.2019.00011

Melke, J., Goubran Botros, H., Chaste, P., Betancur, C., Nygren, G., Anckarsäter, H., Rastam, M., Ståhlberg, O., Gillberg, I. C., Delorme, R., Chabane, N., Mouren-Simeoni, M. C., Fauchereau, F., Durand, C. M., Chevalier, F., Drouot, X., Collet, C., Launay, J. M., Leboyer, M., … Van Maldergem, L. (2008). Abnormal melatonin synthesis in autism spectrum disorders. *Molecular Psychiatry*, *13*(1), 90–98. https://doi.org/10.1038/sj.mp.4002016

Mulder, E. J., Anderson, G. M., Kemperman, R. F. J., Oosterloo-Duinkerken, A., Minderaa, R. B., & Kema, I. P. (2009). Urinary excretion of 5-hydroxyindoleacetic acid, serotonin and 6-sulphatoxymelatonin in normoserotonemic and hyperserotonemic autistic individuals. *Neuropsychobiology*, *61*(1), 27–32. https://doi.org/10.1159/000258640

Nir, I., Meir, D., Zilber, N., Knobler, H., Hadjez, J., & Lerner, Y. (1995). Brief report: Circadian melatonin, thyroid-stimulating hormone, prolactin, and cortisol levels in serum of young adults with autism. *Journal of Autism and Developmental Disorders*, *25*(6), 641–654. https://doi.org/10.1007/BF02178193

Tordjman, S., Anderson, G. M., Bellissant, E., Botbol, M., Charbuy, H., Camus, F., Graignic, R., Kermarrec, S., Fougerou, C., Cohen, D., & Touitou, Y. (2012). Day and nighttime excretion of 6-sulphatoxymelatonin in adolescents and young adults with autistic disorder. *Psychoneuroendocrinology*, *37*(12), 1990–1997. https://doi.org/10.1016/j.psyneuen.2012.04.013

Tordjman, S., Anderson, G. M., Pichard, N., Charbuy, H., & Touitou, Y. (2005). Nocturnal Excretion of 6-Sulphatoxymelatonin in Children and Adolescents with Autistic Disorder. *Biological Psychiatry*, *57*(2), 134–138. https://doi.org/10.1016/j.biopsych.2004.11.003

Table 2: Description of circadian parameters and comparison of circadian parameters between the ASD and control group.

Wrist temperature (ºC)				Motor activity (Acceleration, G/h)				Time in movement (seconds)			
	ASD (n=37)	Control (n=24)	P-value		ASD (n=37)	Control (n=24)	P-value		ASD (n=37)	Control (n=24)	P-value
**Mean**	32.35 ± 0.64	32.58 ± 0.56	NS	Mean	16.22 ± 4.07	19.05 ±4.62	<0.05	Mean	10.42 ±1.48	11.19 ±1.66	<0.05
**IS**	0.48 ± 0.14	0.53 ±0.18	NS	IS	0.34 ± 0.08	0.40 ± 0.10	<0.01	IS	0.53 ± 0.10	0.64 ± 0.11	<0.01
**IV**	0.003 ± 0.002	0.008 ± 0.013	<0.05	IV	0.45 ± 0.09	0.414 ± 0.055	NS	IV	0.22 ± 0.05	0.190 ± 0.049	<0.05
**NRA**	0.42 ±0.14	0.41 ±0.18	NS	NRA	0.61 ± 0.19	0.79 ± 0.23	<0.01	NRA	0.82 ± 0.15	0.92 ± 0.14	<0.01
**M5**	03:39 (01:22-05:30)	03:35 (00:34-05:02)	NS	L5	03:26 (02:24- 04:32)	03:27 (02:25-04:50)	NS	L5	03:04 (02:10- 03:47)	02:59 (02:21 - 03:41)	NS
**VM5**	33.52 ±0.61	33.59 ± 0.54	NS	VL5	2.06 ± 0.55	1.77 ± 0.43	<0.05	VL5	0.58 ± 0.25	0.498 ± 0.143	NS
**L10**	15:17 (13:52-17:02)	15:08 (13:41-16:13)	NS	M10	14:59 (13:51- 15:59)	14:58 (14:17 - 15:38)	NS	M10	15:10 (14:21- 16:03)	14:37 (13:55- 14:57)	NS
**VL10**	31.41 ±0.87	31.53 ±0.94	NS	VM10	26.97 ± 7.87	33.66 ± 9.49	<0.01	VM10	17.10 ± 2.79	19.01 ± 2.99	<0.05
**CFI**	0.60 ±0.08	0.65 ±0.24	NS	CFI	0.65 ± 0.03	0.69 ± 0.04	<0.001	CFI	0.78 ± 0.04	0.83 ± 0.04	<0.01

CFI: circadian function index, IS: inter-daily stability, IV: intradaily variability, NRA: normalized relative amplitude, M5: average measured for the 5 consecutive hours with the maximum values, L10: average measured for the 10 consecutive hours with the minimum values, L5: average measured for the 5 consecutive hours of minimum values, M10: averaged measured for the 10 consecutive hours of maximum values.

Table 3: Description of circadian parameters and comparison of circadian parameters between the ASD and control group.

TAP				Sleep				Total light (log_{10}lux)			
	ASD (n=37)	Control (n=24)	P-value		ASD (n=37)	Control (n=24)	P-value		ASD (n=37)	Control (n=24)	P-value
								Mean	0.81 ± 0.19	1.00 ± 0.13	<0.001
**IS**	0.62 ± 0.13	0.52 ± 0.15	<0.01	IS	0.78 ± 0.07	0.84 ± 0.04	<0.01	IS	0.57 ± 0.12	0.69 ± 0.09	<0.001
**IV**	0.16 ± 0.04	0.14 ± 0.03	NS	IV	0.10 ± 0.02	0.11 ± 0.01	NS	IV	0.06 ± 0.03	0.06 ± 0.01	NS
**NRA**	0.60 ± 0.13	0.52 ± 0.14	<0.05	NRA	0.93 ± 0.04	0.95 ± 0.01	<0.05	NRA	0.47 ± 0.14	0.64 ± 0.09	<0.001
**L5**	03:19 (02:15- 04:27)	03:16 (01:52- 04:40)	NS	M5	03:12 (02:12-04:11)	02:50 (02:21- 03:34)	NS	L5	02:52 (01:53- 03:36)	02:26 (01:27- 03:08)	NS
**VL5**	0.12 ± 0.04	0.16 ± 0.05	<0.01	VM5	0.94 ± 0.04	0.96 ± 0.01	NS	VL5	0.002 ± 0.01	0.000 ± 0.002	NS
**M10**	14:49(13:50-15:57)	14:38 (14:00- 15:17)	NS	L10	14:55 (14:09- 15:42)	14:32 (13:28- 15:24)	NS	M10	14:28 (13:44 – 14:57)	13:56 (13:37- 14:10)	<0.05
**VM10**	0.48 ± 0.06	0.48 ± 0.04	NS	VL10	0.02 ± 0.13	0.002 ± 0.005	NS	VM10	1.53 ± 0.38	1.94 ± 0.27	<0.001
**CFI**	0.45 ± 0.07	0.38 ± 0.10	<0.01	CFI	0.91 ± 0.02	0.92± 0.01	<0.01	CFI	0.84 ± 0.04	0.88 ± 0.03	<0.001

CFI: circadian function index, IS: inter-daily stability, IV: intradaily variability, NRA: normalized relative amplitude, M5: average measured for the 5 consecutive hours with the maximum values, L10: average measured for the 10 consecutive hours with the minimum values, L5: average measured for the 5 consecutive hours of minimum values, M10: averaged measured for the 10 consecutive hours of maximum values, TAP: integrated variable known as thermometry, actimetry and body position.
